# Gold Fillings Sixty-Four Years Old

**Published:** 1880-03

**Authors:** 


Gold Fillings Sixty-Four Years Old.—There is living in Macon a lady who had teeth filled with gold in the year 1815, in the city of Philadelphia, Pa. She was just fourteen years old when the work was done. The fillings are in the crowns of the second upper molars, and are better at this time than many recently put in by dentists.

